# Supplementary material for: Surface Microbiome Profiling of Dental Elevators Using Third‐Generation Sequencing: Implications for Infection Control in Dental Practice
Source: Microbiologyopen. 2025 Dec 8;14(6):e70178. doi: 10.1002/mbo3.70178 (PMC12685768; doi:10.1002/mbo3.70178)
Supplement: Supplementary file 1 — customer_backup. [file MBO3-14-e70178-s001.zip › customer_backup/customer_backup/taxa_summary/krona/samples/M02.Krona.html]

Javascript must be enabled to view this page.

members
magnitude
magnitudeUnassigned

M02.krona

49624

49623

65

65

65

65

65

1

4

19

6

22

3

3

7

7925

60

60

60

60

60

484

484

2

2

2

298

27

27

271

271

184

26

3

5

18

3

3

25

25

84

3

81

46

1

42

3

427

27

26

16

16

10

10

1

1

1

62

22

22

22

9

9

9

2

2

2

8

8

8

17

17

17

4

4

4

23

2

2

2

19

19

19

2

2

2

315

6

6

6

56

56

3

53

190

20

6

14

164

3

161

6

2

4

63

5

5

58

58

2919

1972

5

5

5

772

618

300

3

59

38

183

35

1

1

2

2

93

10

1

82

33

12

4

17

25

25

1195

21

21

1171

1171

3

3

947

640

640

109

508

23

307

1

1

11

11

295

295

3936

1764

1764

117

106

11

1647

18

389

1

404

804

19

1

4

7

3

3

3

3

12

12

12

12

12

12

2

2

10

1

8

1

67

67

1

1

9

6

3

8

8

1

1

23

4

3

3

13

17

9

8

4

4

4

4

771

96

14

5

9

67

9

8

4

4

1

3

2

4

4

1

1

3

1

13

9

9

9

6

6

675

86

86

1

1

163

163

394

387

7

2

2

1

1

28

28

10

10

6

6

4

4

123

63

63

61

2

4

1

1

3

3

56

56

15

18

11

12

89

89

33

33

45

11

10

12

6

6

11

11

164

44

6

6

30

30

8

8

9

1

1

8

8

14

14

8

6

7

7

7

12

12

2

10

78

12

12

21

21

45

45

102

102

2

2

86

2

8

15

9

39

13

5

3

1

1

1

1

8

8

804

31

3

3

3

3

1

1

3

3

18

1

17

3

3

385

173

173

4

4

50

3

47

2

2

49

49

2

2

105

46

35

21

3

82

78

72

6

4

4

271

8

8

16

16

10

10

12

12

1

1

6

6

4

4

189

3

20

166

15

15

2

2

7

7

1

1

1

1

1

34

34

34

15

4

4

4

11

11

11

99

99

99

99

1

53

44

1

79

79

79

79

79

19

16

4

3

10

18

9

195

195

195

195

195

195

717

717

717

139

139

46

93

565

565

42

523

13

13

13

1233

1233

1233

857

10

10

847

810

37

376

376

250

24

44

9

49

12

12

12

5

5

5

2

2

2

5

5

5

160

70

70

1

1

1

38

3

3

35

35

3

3

3

6

1

1

5

5

22

17

17

5

5

90

90

15

15

15

16

16

16

4

4

4

10

10

10

45

45

45

12

12

12

12

5

2

3

7

7

196

165

165

1

1

1

164

115

113

2

34

3

30

1

15

15

24

24

1

1

1

23

23

23

7

7

7

7

7

370

3

3

3

3

3

367

367

8

8

8

24

24

24

1

1

1

291

35

35

16

11

5

55

55

131

131

54

54

42

42

42

1

1

1

457

209

14

14

14

14

195

74

57

52

5

2

2

3

3

12

12

121

3

3

36

36

6

6

76

76

120

83

83

83

32

35

13

3

3

3

3

3

34

34

2

2

14

2

12

8

6

2

10

5

2

3

8

1

1

1

1

7

7

7

7

17

17

17

17

3

1

5

8

103

103

5

5

5

77

77

77

21

21

21

1

1

1

1

1

1

225

1

1

1

1

1

45

45

45

2

2

20

20

6

6

17

8

9

179

179

179

11

10

1

156

54

102

6

4

2

6

6

625

625

625

625

71

71

554

147

365

42

460

96

96

96

96

96

2

2

2

2

2

362

219

208

184

39

132

13

24

4

16

4

11

11

10

1

12

10

10

10

2

2

2

124

3

3

3

121

121

21

100

7

7

1

1

6

1

5

14

14

14

9

9

9

5

5

2

1

2

37

24

11

11

4

4

7

5

2

13

12

12

12

1

1

1

11

11

11

11

11

2

1

1

1

1

1

1

1

1

9680

4375

76

1

1

1

74

2

2

4

4

68

3

65

1

1

1

150

150

83

1

82

58

58

9

9

41

41

1

1

1

1

37

37

2

2

60

60

1

1

59

6

53

1

1

1

1

451

451

30

1

1

3

18

7

19

19

3

3

50

1

2

30

4

1

12

99

4

58

26

11

3

3

212

4

17

1

2

49

11

1

4

8

2

20

5

43

21

1

9

8

2

4

35

10

6

19

1365

1365

3

1

2

1259

1259

9

4

2

1

2

46

8

22

16

2

2

40

19

19

2

1

1

5

5

1808

61

28

28

33

8

5

20

29

29

29

52

3

2

1

41

4

1

5

2

27

2

8

8

5

5

5

3

3

3

2

2

2

79

16

2

3

11

63

8

49

6

5

5

5

66

57

57

9

9

447

12

5

7

45

45

138

138

6

5

1

13

13

1

1

6

6

2

2

224

14

210

297

1

1

114

106

8

3

3

3

3

98

1

76

5

13

1

2

78

4

1

13

10

3

3

3

32

9

96

35

35

3

3

33

33

21

21

4

4

56

56

2

54

610

179

179

1

1

1

1

13

13

315

14

44

6

1

90

1

14

131

8

6

33

9

14

10

12

12

56

56

4

4

4

4

61

8

6

6

2

2

53

7

4

3

15

1

6

3

5

6

1

1

4

11

11

14

14

3

3

3

3

12

3

3

3

1

1

1

2

2

2

1

1

1

2

2

2

3

3

3

5

5

5

5

338

338

1

1

14

13

1

1

1

29

2

27

22

1

21

271

35

69

167

5305

11

11

11

11

1590

6

5

5

1

1

3

3

3

994

959

959

5

5

22

22

8

8

21

21

21

161

32

20

12

129

109

20

32

32

32

373

373

373

3308

14

14

14

27

27

27

52

52

52

1773

4

4

1

1

1581

1581

84

22

10

33

19

103

2

34

1

9

7

6

33

11

39

13

13

1

1

4

1

1

2

19

19

2

2

2

2

2

58

1

1

18

18

38

38

1

1

144

35

35

31

11

20

78

78

64

64

64

1056

3

3

7

7

18

18

31

1

30

41

7

17

17

5

4

1

34

20

14

31

2

29

5

5

3

3

840

840

37

37

1

1

79

22

22

57

57

91

69

35

27

8

32

32

2

2

22

22

22

56

56

56

1

43

12

53

53

53

53

24

24

24

24

48

48

48

48

1

1

1

1

16

16

16

11

3

2

43

14

1

1

3

3

4

4

5

5

1

1

29

1

1

2

1

1

20

10

10

6

3

2

1

64

1

1

1

13

13

13

7

7

6

1

23

22

22

1

1

20

1

1

19

9

10

1

1

1

1

1

1

256

154

154

154

21

21

8

8

1

1

10

10

5

5

19

8

11

90

89

1

102

1

1

1

1

41

20

5

5

2

1

1

13

3

10

1

1

1

10

10

9

1

10

4

4

6

6

60

60

60

2

1

29

28

27

27

27

27

27

5

9

1

12

22516

6408

13

13

2

2

6

1

1

4

5

5

19

19

12

12

7

7

159

5

5

5

2

2

2

152

72

72

80

80

14

14

14

3

11

3126

3122

13

12

1

45

5

40

58

44

14

5

5

1

1

29

29

5

5

6

6

8

8

130

13

64

2

1

20

8

1

9

6

3

1

2

26

26

4

1

3

892

353

1

32

504

2

12

10

2

94

37

55

1

1

481

37

438

6

17

17

30

30

106

106

2

2

7

7

1

1

29

29

70

70

24

24

125

1

3

36

85

34

34

6

6

13

13

21

10

11

11

4

7

24

24

771

14

116

2

8

3

41

510

5

27

45

22

22

4

4

4

2248

1383

561

561

76

6

16

54

237

237

1

1

178

178

316

275

4

5

32

14

14

5

5

5

689

14

14

624

624

18

18

33

33

171

12

12

10

10

149

149

22

22

22

5

1

2

12

2

41

8

8

8

2

2

2

31

31

31

1

1

1

1

22

11

11

11

11

11

11

743

349

11

11

22

22

26

19

2

3

2

6

1

5

131

131

1

1

16

9

7

2

2

7

7

16

16

45

45

66

1

1

64

69

69

69

6

6

6

15

2

2

5

1

4

8

8

2

2

2

1

1

1

7

3

3

4

4

294

13

6

7

55

14

13

10

18

137

136

1

63

5

4

1

50

3

22

22

4

2

2

11909

1067

767

767

24

1

23

667

18

4

17

11

2

300

9

9

8

8

22

22

33

27

1

5

139

9

130

17

17

68

68

4

4

1187

356

97

97

193

98

87

8

2

2

57

35

22

7

7

831

243

198

45

6

6

13

13

3

3

66

66

10

10

120

20

100

151

151

31

31

131

46

85

26

26

23

23

8

8

326

63

1

1

4

4

58

11

47

263

263

261

2

148

148

139

3

5

5

6

18

2

13

2

8

70

7

3

3

6

4

2

367

367

367

367

12

2

2

2

8

8

8

2

2

2

8777

6

6

2

4

168

5

5

162

162

1

1

7928

7924

1188

89

134

5060

257

889

38

269

4

4

13

13

13

606

10

10

3

3

5

3

2

1

1

267

51

1

215

2

2

234

176

58

6

6

71

2

69

4

2

2

2

2

1

1

56

56

56

20

20

20

19

1

5

5

5

5

2

2

2

2

2

4197

14

14

14

14

4183

1878

19

9

10

1859

1007

252

167

19

84

179

151

2290

2104

2104

128

128

58

3

55

15

15

15

53

6

3

3

3

3

1

1

1

1

2

2

2

2

2

2

2

2

2

18

1

1

1

1

8

8

1

1

7

7

4

4

4

4

5

5

5

5

27

27

27

27

27

38

38

38

38

38

25

13

6

6

6

6

6

5

1

68

49

49

46

46

46

3

3

3

19

19

19

10

8

2

7

7

2

1

1

4104

4101

276

4

4

4

269

2

1

1

65

11

15

39

54

54

6

2

2

2

6

6

79

11

10

4

10

8

22

3

10

1

18

3

1

8

5

1

1

1

4

4

5

5

18

18

3

3

8

8

3

3

1

2

24

4

4

4

18

18

12

6

2

2

2

3629

928

34

34

38

38

126

126

730

730

152

10

10

26

26

116

6

2

3

40

44

6

15

58

58

58

136

3

3

133

8

22

103

217

217

184

6

21

6

768

768

113

450

110

1

3

6

72

13

471

56

56

137

101

36

278

79

60

43

50

46

883

1

1

489

489

31

31

77

77

149

17

69

12

12

39

136

10

8

41

25

19

5

28

16

16

16

17

8

6

6

2

2

9

9

9

155

26

4

4

20

3

15

2

2

2

26

2

2

15

3

12

7

7

2

2

16

16

9

7

4

4

4

28

6

6

7

7

15

15

55

48

1

8

8

17

13

1

2

1

1

5

5

1

1

1

1

1

2

2

2

2

2

91

91

91

11

11

11

13

13

13

67

67

67

1

1

1

1

1

1

1
